# Croatia is a safe tourist destination – study of foreign citizen mortality in Splitsko-dalmatinska and Primorsko-goranska County during the period 2001-2010

**DOI:** 10.3325/cmj.2013.54.291

**Published:** 2013-06

**Authors:** Kristijan Bečić, Darija Jandrić Bečić, Morana Čengija, Goran Ćurić, Antonio Alujević, Marija Definis-Gojanović

**Affiliations:** 1 University of Split School of Medicine, Split, Croatia; 2 University of Rijeka School of Medicine, Rijeka, Croatia; 3 DNA Laboratory, School of Medicine, J. J. Strossmayer University, Osijek, Croatia; 4 Clinical Hospital Centre Split, Split, Croatia

## Abstract

**Aim:**

To investigate the mortality rate of foreign citizens in Croatia.

**Methods:**

Data were collected from the Departments for Forensic Pathology in Split and Rijeka, which are the autopsy centers of the counties with approximately 35% of total foreign visitors, as well as from the Croatian Central Bureau for Statistics for the period 2001-2010. The mortality rate (number of deaths of members of each nationality per 100 000 entrances ratio) and standardized mortality ratio (ratio between the observed and expected number of deaths) were calculated, and χ^2^ goodness of fit test was used for statistical analyses.

**Results:**

There were 447 deaths (325 men, 72.7%) of foreign citizens (mortality rate of 0.0015%). A total of 207 deaths (46.3%) were by natural causes, more often among older people, and 240 deaths (53.7%) were injury deaths, more often among younger people, mostly by drowning or traffic-related (22.2% and 18.6% of all deaths, respectively). Most represented were citizens of German, Austrian, Czech, and Italian nationality, with 115 (25.7%), 59 (13.2%), 58 (13.0%), and 52 deaths (11.6%), respectively. Mortality rate by nationality showed no significant difference (*P* < 0.05). The standardized mortality ratio was lowest in Hungarian and Czech citizens (0.17) and highest in US citizens (0.35).

**Conclusions:**

Croatia has low foreign citizens’ mortality rate and could be considered a safe tourist destination.

In the period 2001-2010, the number of foreign citizens who visited Croatia rose from 6 544 217 to 10 604 116, twice the Croatian population every year. As it is impossible to distinguish tourists from visitors who come to Croatia for business-related reasons, the term “foreign citizens” was used through the article. In the 10-year period, the number of entries in Croatia was 84 595 522, and 35.9% (30 357 901) entered Splitsko-dalmatinska and Primorsko-goranska Counties (1). Tourism amounts to 13.6% of Croatian GDP (2010) (2), so health and safety of foreign citizens are an important issue. Croatia has been chosen for the top tourist destination in the world several times (by Lonely Planet for 2005, by National Geographic Adventure Magazine for 2006, by New York Times for 2012). The country has very high hygienic standards, good health care, excellent drinking tap water, virtually no dangerous infectious diseases, and a small number of dangerous species.

Nearly 50% of travelers worldwide suffer from some kind of illness (3-6), and commonly these conditions are nothing more than gastrointestinal problems (like travelers’ diarrhea), which are not dangerous and are rarely reported as a cause of death (3,7). However, some foreign citizens arrive with more serious conditions, mostly cardiovascular in nature, and they are usually unaware of them. Cardiovascular diseases are the single most common cause of death among this population, while the majority of unintentional deaths are ascribed to traffic related deaths and drowning (4,8,9). The aim of this study was to investigate the mortality rate of foreign citizens in Splitsko-dalmatinska and Primorsko-goranska Counties.

## Materials and methods

Data were collected from autopsy records of Clinical Departments for Forensic Pathology, Split and Rijeka and from the Statistical Yearbooks of the Croatian Central Bureau for Statistics (CBS). From autopsy records, we obtained the foreign citizen-related data (sex, age, country of origin, cause and type of death). From the CBS, we obtained the number of foreign citizens who entered Croatia in the investigated period, the number of foreign citizens who entered the analyzed region, nationality distribution, and the contribution of tourism to the Croatian GDP. Mortality rate (number of deaths of members of each nationality per 100 000 entries of that nationality) and standardized mortality ratio (the ratio between the observed and expected number of deaths per unit of time) were calculated using the data only for the period 2005-2010, because the distribution of nationalities by counties was not available for previous years. χ^2^ goodness of fit was used for comparison of mortality rates between the countries. Statistical significance level was *P* < 0.05. National mortality rates from WHO database (10) were used for mortality ratio comparisons and standardized mortality ratio calculations. As WHO data on mortality were annual death rates and average time of foreign citizens’ stay in Croatia was 5.6 days (1), overall mortality rates were adjusted to a 5.6-day period. To do so, we assumed that mortality rates in reference populations were constant throughout the year.

## Results

**Figure 1:**
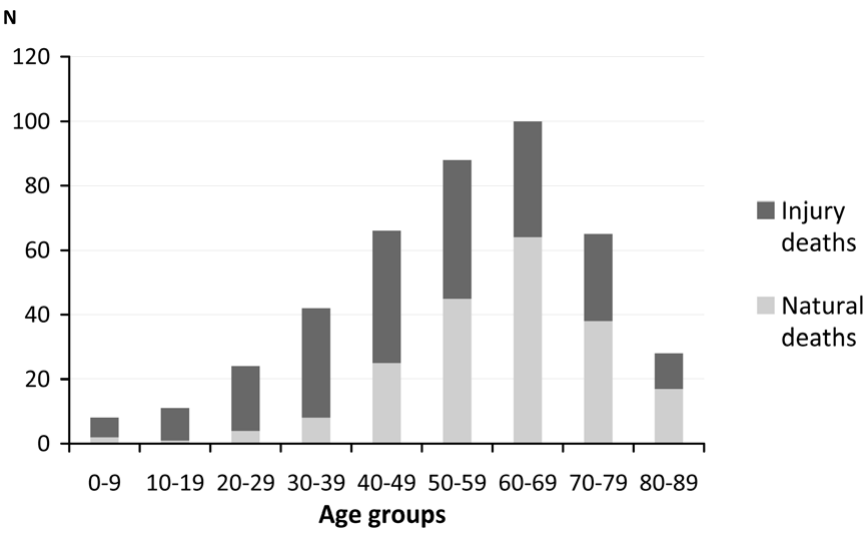
Age, number of deaths, and cause of death among foreign citizens in Splitsko-dalmatinska and Primorsko-goranska County in the period 2001-2010.

The number of foreign citizens who entered the analyzed region increased from 2 398 811 2001 to 3 788 774 in 2010, ie, by 57.9%, while the number of deaths decreased by 11.9%, from 42 in 2001 to 37 in 2010. The number of foreign citizens who entered the analyzed region in the period was 30 357 901. There were 447 deaths of foreign visitors (1.47 per 100 000, 325 men, 72.7%). The majority of deaths were in the age-group between 40 and 79 years (median age 54 years), with the greatest number of deaths in the age group 60-69 years (100 tourists; 23.2%, Figure 1).

Approximately two thirds of the deceased were from four countries (Table 1): Germany, Austria, Czech Republic, and Italy (25.7%, 13.2%, 13.0%, and 11.6%, respectively). The majority of deaths were by natural causes (46.3%), particularly from heart diseases (85.3% of natural and 38.93% of total deaths). There were 240 injury deaths (0.8 per 100 000), 229 of which were unintentional deaths, mostly drowning and traffic-related. A total of 99 (22.2%; 0.33 per 100 000) deaths occurred by drowning and 83 (18.6%; 0.27 per 100 000) were traffic-related. Among intentional deaths, there were only 3 homicides (0.7%) and 8 suicides (1.8%).

**Table 1:** Deaths of foreign citizens by countries and cause of death in Splitsko-dalmatinska and Primorsko-goranska County in the period 2001-2010

	Natural deaths	Injury deaths							Total (%)
		drowning	traffic	fall from height	suicide	homicide	other injury deaths	total injury deaths	
Germany	57	32	18	4	1	0	3	58	115 (25.7)
Austria	30	15	8	2	1	0	3	29	59 (13.2)
Czech Republic	18	21	12	3	1	0	3	40	58 (13.0)
Italy	23	5	14	3	2	2	3	29	52 (11.6)
Hungary	13	6	2	0	0	0	3	11	24 (5.4)
Poland	10	4	3	4	0	1	2	14	24 (5.4)
Slovakia	4	1	15	0	1	0	1	18	22 (4.9)
France	5	5	2	1	0	0	0	8	13 (2.9)
UK	6	1	2	1	1	0	0	5	11 (2.5)
USA	5	–	–	2	0	0	2	4	9 (2.0)
Other	36	9	7	0	1	0	7	24	60 (13.4)
Total (%)	207 (46.3)	99 (22.2)	83 (18.6)	20 (4.5)	8 (1.8)	3 (0.7)	27 (6.0)	240 (53.7)	447 (100)

The highest mortality rate was found in foreign citizens from Slovakia (2.79) and the lowest in Italian citizens (1.29) (Table 2). However, the difference in mortality rates between the nationalities was not statistically significant (*P* > 0.05). The highest standardized mortality ratio was found in US citizens (0.35) and the lowest in Czech and Hungarian citizens (0.17) (Table 2).

**Table 2:** Standardized mortality rates of foreign citizens by country in the period 2001-2010

	Mortality rate for the country*	Number of entrances	Number of deaths	Expected number of deaths	Standardized mortality ratio	Mortality rate of foreign citizens per nationality in presented population sample
Croatia	8.73					
Germany	6.31	3 209 177	51	202.50	0.25	1.59
Austria	6.01	1 673 276	29	100.56	0.29	1.73
Czech Republic	7.95	1 786 042	24	141.99	0.17	1.34
Italy	5.34	2 484 407	32	132.67	0.24	1.29
Hungary	10.03	966 037	16	96.90	0.17	1.66
Poland	8.99	911 826	15	81.97	0.18	1.65
Slovakia	9.36	787 928	22	73.75	0.30	2.80
France	5.66	829 444	11	46.94	0.23	1.33
UK	6.43	436 760	8	28.08	0.28	1.83
USA	7.46	271 512	7	20.25	0.35	2.58
Netherlands	6.03	355 982	5	21.47	0.23	1.41

*WHO mortality rate for citizens of each country in their homeland (10).

## Discussion

Some foreign embassies recommend Croatia as a relatively safe country with low levels of crime (11,12). This study confirmed such claims and found a low foreign citizens’ mortality rate in Croatia. Studies on foreign citizens’ mortality in other countries are limited (4,5,13), while there are more studies on the mortality of citizens while abroad (8,9,14).

The single most usual cause of deaths of foreign citizens in our study was ischemic heart disease (38.9%), which is in line with previous studies (5,8,9,13-16). Since preventive measures can do very little when deaths by natural causes are concerned, such deaths are a poor marker of travelers’ safety. However, while Croatia cannot offer preventive care for chronic diseases to foreign citizens, prompt treatment could be provided. It can be stated that the work of the Croatian Emergency Medical Services is more than satisfactory, especially after its reorganization in the period 2006-2011, when emergency medicine specialization for physicians and additional specialized training for nurses/technicians, integrated emergency admission areas in hospitals, and telemedicine were introduced, and guidelines and algorithms for care were created (17).· However, for all travelers with chronic or underlying diseases it is highly recommended to seek pre-travel medical consultation (18).

A better marker of safety is the number of injury deaths, as all injury deaths, including suicides and homicides, should be considered preventable. The majority of injury deaths was in the form of unintentional deaths. Drowning mortality rate was 0.33 per 100 000 tourists (22.2% of all deaths), which is not high when compared to Australian data, where crude beach surfing drowning rate is 2.6 per 100 000 (25% of all drowning deaths) (19). At the same time, Leggat and Wilks reported only 5% of drowning deaths in tourists visiting Australia (4). Several studies report drowning as the second most frequent cause of death in foreign citizens (4,18,20). As most deaths occur close to the shore, it is important to have lifeguards on major beaches. It is difficult to educate all foreign citizens on the dangers of the open sea, but Ministries of Tourism and Justice should enact more rigorous laws of deep water diving and sailing, in order to prevent participation of inexperienced people in these activities.

The traffic-related mortality rate was 0.27 per 100 000 tourists (18.6% of all deaths), which is not high when compared to traffic mortality of Croatian citizens of 11.45 per 100 000 in 2011 (21). Traffic accidents were the leading cause of injury deaths in foreign citizens visiting the USA (37%) and Australia (14%) (4,20). There are numerous measures to reduce traffic mortality, and Croatian authorities have focused on building highways, redesigning bad intersections and bad roads, and especially on getting drunk drivers off the roads. This also disputes the theory by Sumala (22) and Petridou et al (23) that foreign citizens are at larger risk of traffic accidents than domestic drivers.

Suicides and homicides were very rare in studied period. The homicide death rate of 0.7% is lower than 0.8% reported in Australia (4). This difference is even more evident if we compare the crude death rates of 0.1 per million in Croatia and 0.9 per million in Australia (24). When we compare it with the crude death rate of Croatian citizens, which is 1.14 per 100 000, it is evident that foreign citizens are in less danger to be murdered than Croatian citizens (21). The measures that are in place to prevent such deaths in the Croatian population serve the same purpose for foreign citizens.

When we exclude deaths by natural causes as an insignificant marker of tourist safety, in our sample the injury death rate was 0.8 per 100 000, which is 2.5 times lower than the Australian estimate (2.01 per 100 000).

Due to a greater number of travelers from some countries, some nationalities might seem more at risk, and therefore, we calculated mortality rates for each nationality. Although it may seem that Slovak citizens are in the lead, it is because most of Slovak citizens (14/22) lost their lives in a single bus accident. Standardized mortality ratios for all nationalities were lower than expected or previously reported (5). However, these results should be taken with caution as demographic data of the population that travels is not the same as the general population usually taken for mortality rate calculations. Also, we have to mention the problem of adjusting the mortality rates. As Croatia is a tourist destination, the most of tourists arrive in the same time, increasing the risk at one period in the year. Another limitation that has to be taken into account is the fact that each nationality has different proportions of death causes in the homeland and abroad.

In conclusion, Croatia is a safe traveling destination. Its injury death mortality rate is only 0.8 per 100 000 foreign citizens’ entries, mostly due to drowning and traffic-related deaths. Although these deaths occur suddenly and unexpectedly, the number could be reduced even more if safety information about the dangers of the open sea was prepared and more strict traffic, diving, and sailing regulations were introduced.
